# Optimising Extracellular Vesicle Metabolomic Methodology for Prostate Cancer Biomarker Discovery

**DOI:** 10.3390/metabo14070367

**Published:** 2024-06-28

**Authors:** Mahmoud Assem Hamed, Valerie Wasinger, Qi Wang, Joanna Biazik, Peter Graham, David Malouf, Joseph Bucci, Yong Li

**Affiliations:** 1 St. George and Sutherland Clinical Campuses, School of Clinical Medicine, University of New South Wales (UNSW) Sydney, Kensington, NSW 2052, Australia; assem.hamed@unsw.edu.au (M.A.H.); qi.wang18@unsw.edu.au (Q.W.); peter.graham@health.nsw.gov.au (P.G.); joseph.bucci@health.nsw.gov.au (J.B.); 2 Cancer Care Centre, St. George Hospital, Kogarah, NSW 2217, Australia; 3 Bioanalytical Mass Spectrometry Facility, Mark Wainwright Analytical Centre, University of New South Wales (UNSW) Sydney, Kensington, NSW 2052, Australia; v.wasinger@unsw.edu.au; 4 Electron Microscope Unit, Mark Wainwright Analytical Centre, University of New South Wales (UNSW) Sydney, Kensington, NSW 2052, Australia; joanna.richmond@unsw.edu.au; 5 Department of Urology, St. George Hospital, Kogarah, NSW 2217, Australia; david@drdavidmalouf.com.au

**Keywords:** prostate cancer, extracellular vesicles, metabolomics, metabolite extraction, chromatography columns, biomarker discovery

## Abstract

Conventional diagnostic tools for prostate cancer (PCa), such as prostate-specific antigen (PSA), transrectal ultrasound (TRUS), digital rectal examination (DRE), and tissue biopsy face, limitations in individual risk stratification due to invasiveness or reliability issues. Liquid biopsy is a less invasive and more accurate alternative. Metabolomic analysis of extracellular vesicles (EVs) holds a promise for detecting non-genetic alterations and biomarkers in PCa diagnosis and risk assessment. The current research gap in PCa lies in the lack of accurate biomarkers for early diagnosis and real-time monitoring of cancer progression or metastasis. Establishing a suitable approach for observing dynamic EV metabolic alterations that often occur earlier than being detectable by other omics technologies makes metabolomics valuable for early diagnosis and monitoring of PCa. Using four distinct metabolite extraction approaches, the metabolite cargo of PC3-derived large extracellular vesicles (lEVs) was evaluated using a combination of methanol, cell shearing using microbeads, and size exclusion filtration, as well as two fractionation chemistries (pHILIC and C18 chromatography) that are also examined. The unfiltered methanol–microbeads approach (MB-UF), followed by pHILIC LC-MS/MS for EV metabolite extraction and analysis, is effective. Identified metabolites such as L-glutamic acid, pyruvic acid, lactic acid, and methylmalonic acid have important links to PCa and are discussed. Our study, for the first time, has comprehensively evaluated the extraction and separation methods with a view to downstream sample integrity across omics platforms, and it presents an optimised protocol for EV metabolomics in PCa biomarker discovery.

## 1. Introduction

Current diagnostic methods for prostate cancer (PCa) include serum prostate-specific antigen (PSA), transrectal ultrasound (TRUS), digital rectal examination (DRE), magnetic resonance imaging (MRI), and tissue biopsy. PSA has limitations in distinguishing between benign prostate hyperplasia (BPH) and indolent PCa [1]. TRUS is invasive and can cause side effects like impotence and pain [2]. Tissue biopsies can give false negatives due to PCa’s complexity and multifocality [3]. Better biomarkers are needed to reduce unnecessary biopsies and overtreatment. To overcome these challenges, two key considerations are crucial. Firstly, replacing invasive tissue biopsies with less invasive methods like liquid biopsy (LB) shows promise. Secondly, advanced molecular technologies such as genomics, proteomics, and metabolomics can effectively address tumour heterogeneity. These methods can comprehensively profile tumours, identifying robust biomarkers. This can improve risk assessment and personalise medical interventions through tailored medicine.

LB is gaining attention as a non-invasive method with various roles in cancer management. It aids in early cancer detection, selecting patients for surgical biopsy, monitoring low-risk cancer, and tracking disease recurrence post-treatment. This progress enables tailored treatment strategies for PCa patients [4]. Extracellular vesicles (EVs), lipid-bilayer nanovesicles secreted by various cell types into varied body fluids, play essential roles in intercellular communication [5]. Researchers have utilised various molecular technologies, including omics platforms, to explore the molecular cargo carried by EVs in clinical settings. Metabolomics, unlike genomics and proteomics, offers insights into environmental influences. This characteristic makes metabolomics adept at detecting non-genetic factors, improving cancer biomarker discovery for early diagnosis and disease monitoring [6]. Thus, there is a genuine need for a robust methodological framework to accelerate efforts in metabolome profiling for PCa biomarker discovery [7].

Despite numerous previous metabolomic studies employing various metabolite extraction methods for EVs from different sources [8,9,10], no single standardised protocol was achieved. This study aimed to address this gap by optimising an effective protocol for metabolite extraction from EVs, followed by the evaluation of results using different chromatography separation techniques. In the field of EV metabolomics, our recent literature review has summarised diverse metabolite extraction methods and chromatography columns used for metabolomic analysis and has shown a lack of prior research focused on investigating new approaches [11]. In this study, large extracellular vesicles (lEVs) isolated from a PC3 PCa cell line were employed, aiming to optimise an EV metabolomic methodology for the purpose of biomarker discovery in PCa diagnosis and risk stratification.

## 2. Methods and Experimental Design

### 2.1. Preparation for lEVs Isolation from Cell Culture Medium (CCM)

The PC3 cell line, sourced from the American Type Culture Collection (ATCC, Manassas, VA, USA) under the designation (ATCC^®^CRL-1435TM), was cultured in RMPI 1640 medium and maintained in a 37 °C, 5% CO_2_ cell incubator until reaching 60–70% confluency. Subsequently, cells were rinsed with DPBS and incubated in exosome-depleted medium for 48 h. CCM was then collected for lEV isolation, undergoing centrifugation steps at 300× *g* for 5 min to remove cellular debris, followed by 2000× *g* for 20 min, and then 10,000× *g* for 30 min at 4 °C. The resulting pellets were identified as lEVs, while the supernatant was filtered through a 0.22 µm filter and subjected to ultracentrifugation to collect sEVs. The PC3 cell line used is negative for mycoplasma testing and was authenticated within the last three years through Short Tandem Repeat (STR) profiling by employing the PowerPlex^R^ 18D System (Promega, Madison, WI, USA).

### 2.2. Nanoparticle Tracking Analysis (NTA)

NTA was employed to assess the particle size distribution and concentration of both lEVs and sEVs isolates. NTA was conducted using the NanoSight NS300 system (NanoSight Technology, Malvern, Worcestershire, UK).

### 2.3. Transmission Electron Microscopy (TEM)

TEM analysis was performed utilising a JEOL 1400 microscope with a voltage of 100 kV. The magnification power ranged from 80,000× to 100,000× (100 nm to 200 nm). Then, 10 µL of each sample was applied to the grid and left to absorb for 10 min at room temperature (RT). Following absorption, the samples underwent negative staining by exposure to a filtered 2% aqueous solution of uranyl acetate.

### 2.4. Western Blot (WB)

The EVs pellets, obtained through isolation, were lysed using RIPA buffer (Thermo Scientific, Waltham, MA, USA), along with proteinase and phosphatase inhibitor cocktail (PIC) (100×) (Thermo Scientific). The protein concentration in the lysed samples was quantified using bicinchoninic acid (BCA) assay. To assess the expression of lEVs and sEVs markers in each isolate, 10 µg of protein samples was loaded onto individual gel lanes. All antibodies were utilised at a dilution ratio of 1:2000, except for calnexin which was diluted at 1:1000. A detailed description of the lysis process, BCA assay, WB procedures, and antibodies utilised in this study is included in Supplementary Materials.

### 2.5. Metabolite Extraction

**Figure 1:**
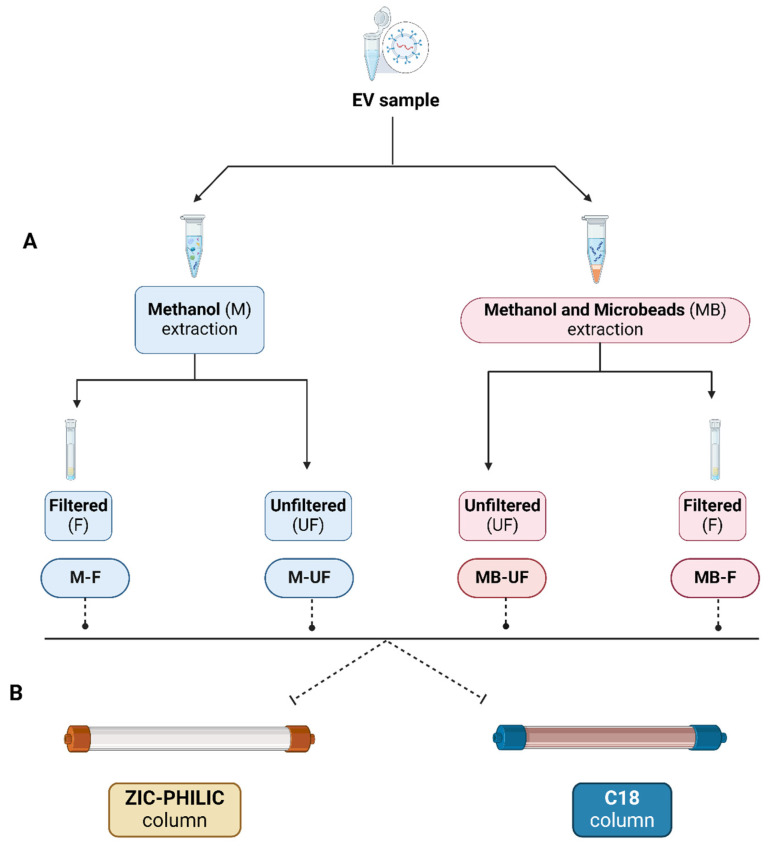
The workflow of metabolite extraction from lEV followed by compound separation for metabolomic analysis. (**A**) Metabolite extraction: four different approaches were applied, including M-F, M-UF, MB-F, and MB-UF. In the M-UF approach, EV samples were treated with 80% methanol, followed by centrifugation, and drying of the supernatant for metabolomic analysis. The MB-UF method involved additional steps of bead-beating and subsequent removal of microbeads before the same centrifugation and drying process. In the M-F and MB-F approaches, after the initial centrifugation, the supernatant underwent additional filtration using centrifugal filter microtubes before the final drying step. All samples were stored at −80 °C post-extraction. (**B**) Compound separation: after extracting metabolites using four different approaches, samples were subjected to liquid chromatography–mass spectrometry (LC–MS) using two different columns separately (pHILIC and C18).

Metabolite extraction from EV samples was conducted using four distinct approaches: (1) methanol-unfiltered (M-UF), (2) methanol and beads-unfiltered (MB-UF), (3) methanol-filtered (M-F), and (4) methanol and beads-filtered (MB-F). Figure 1 illustrates a schematic workflow for the process of metabolite extraction. To eliminate the variability that may arise from four different methodologies, we utilised the same EV subtype isolated from a single cell line. In this study, lEVs were isolated from the PC3 cell line and three replicates were tested for each metabolite extraction approach. Four distinct metabolite extraction approaches were evaluated separately using two different chromatography separation columns: pHILIC and C18. The detailed procedures run in this process are as follows:

1 M-UF approach:

EV samples were treated with 80% methanol (final concentration *v*/*v*). The samples were centrifuged at 11,000× *g* for 45 min at 4 °C. Following this step of centrifugation, the supernatant was collected in glass vials and was dried out using a vacuum centrifuge for 40 min. The dry metabolite-containing samples (in glass vials) were then stored at −80 °C for metabolomic analysis.

2 MB-UF approach:

EV samples were treated with 80% methanol (final concentration *v*/*v*) and 0.1 g of microbeads [Zirconium (0.1 mm diameter)]/100 µL of lysis solution in an Eppendorf. The samples were subjected to a bead-beater for four cycles of 40 s with 2-min intervals on ice. The samples were centrifuged at 11,000× *g* for 45 min at 4 °C to eliminate microbeads. Following this, the samples were centrifuged at 11,000× *g* for 45 min at 4 °C. Following this step of centrifugation, the supernatant was collected in glass vials and was dried out using a vacuum centrifuge for 40 min. The dry metabolite-containing samples (in glass vials) were then stored at −80 °C for metabolomic analysis.

3 M-F approach:

EV samples were treated with 80% methanol (final concentration *v*/*v*). The samples were centrifuged at 11,000× *g* for 45 min at 4 °C. Following this step of centrifugation, the supernatant was separated and underwent an additional centrifugation round at 11,000× *g* for 45 min at 4 °C using centrifugal filter microtubes Microcon^®^-3K Ultracel YM-3 (MILIPORE, Bedford, MA, USA). The supernatant was then collected in glass vials and was dried out using a vacuum centrifuge for 40 min. The dry metabolite-containing samples (in glass vials) were then stored at −80 °C for metabolomic analysis.

4 MB-F approach:

EV samples were treated with 80% methanol (final concentration *v*/*v*) and 0.1 g of microbeads [Zirconium (0.1 mm diameter)]/100 µL of lysis solution in an Eppendorf. The samples were subjected to a bead-beater for four cycles of 40 s with 2-min intervals on ice. The samples were centrifuged at 11,000× *g* for 45 min at 4 °C to eliminate microbeads. Following this, the samples were centrifuged at 11,000× *g* for 45 min at 4 °C. Following this step of centrifugation, the supernatant was separated and underwent an additional centrifugation round at 11,000× *g* for 45 min at 4 °C using centrifugal filter microtubes Microcon^®^-3K Ultracel YM-3 (MILIPORE, Bedford, MA, USA). The supernatant was then collected in glass vials and was dried out using a vacuum centrifuge for 40 min. The dry metabolite-containing samples (in glass vials) were then stored at −80 °C for metabolomic analysis.

### 2.6. Global Metabolomics: Using ZIC-pHILIC and C18 Chromatography

Two different separation columns (ZIC-pHILIC and C18) were employed in this study. In the case of the ZIC-pHILIC column (to be referred as pHILIC), dry samples prepared via four different extraction approaches were resuspended in 50 µL of CH_3_CN:H_2_O (9:1) (*v*/*v*) (PH 5.5), while samples were reconstituted in 50 µL of 0.1 formic acid (pH 3) to be tested by the C18 column. Metabolomic analysis was conducted using an ultra-high performance liquid chromatography (UHPLC) system with a Q-Exactive HF mass spectrometer (Thermo Electron, Waltham, MA, United States). A detailed description is included in Supplementary Materials.

### 2.7. Statistical Analysis

Statistical analysis involved unpaired *t*-tests using GraphPad Prism 0.7 software. Error bars denoted ± standard error of the mean (SEM), with significance levels marked as * *p* < 0.05, ** *p* < 0.01, *** *p* < 0.001, and **** *p* < 0.0001. Venn diagrams were created with Funrish 3.1.4 software. Ingenuity Pathway Analysis (IPA) QIAGEN software was used to explore metabolic pathways and biological functions of EV metabolites from a PCa cell line. Raw data were compared against the Human Metabolome Database (HMDB), Chemical Abstract Services (CAS), Kyoto Encyclopedia of Genes and Genomes (KEGG), and ChemSpider databases for metabolite annotation.

## 3. Results and Discussion

### 3.1. Confirmation and Characterisation of EVs Isolated from PC3 Cell Line

This study characterised EV subtypes using NTA, TEM, and WB analyses. NTA determined average sizes and concentrations of sEVs and lEVs, with mean sizes of 151 nm and 252 nm, respectively, consistent with minimal information for studies of extracellular vesicles 2023 (MISEV2023) guidelines [12]. Concentrations of isolated lEVs (2.7 × 10^9^ particles/mL) were suitable for metabolomic analysis, meeting requirements established in previous studies by Puhka et al. in 2017 [13] and Altadill et al. in 2016 [14] (Supplementary Materials, Figure S1A). Using TEM, both sEVs and lEVs displayed the characteristic cup-shaped morphology typical of EVs. Although there was notable variability in EV dimensions across various captured fields, an average size and typical morphology of EVs were consistently depicted (Supplementary Materials, Figure S1B). These observations serve to validate the NTA results, confirming the quality of the isolated EV preparations. EV quantity was assessed through BCA assay, indicating successful metabolomic potential when protein content ranged between 90 and 100 µg. WB analysis targeting calnexin ensured the absence of intracellular contamination, while specific EV biomarkers (CD81, CD63, syntenin, flotillin-1, and HSP70) confirmed EV exclusivity. The absence of calnexin in both lEVs and sEVs, detected only in cell lysate, indicated a lack of contamination with endoplasmic reticulum or cytoskeletal components, consistent with MISEV2018 guidelines [15] and previous research by Hosseini-Beheshti et al. [16] (Supplementary Materials, Figure S1C). Collectively, the confirmation tests including NTA, TEM, and WB affirmed the purity and quality of isolated lEVs for subsequent metabolomic analysis.

### 3.2. Analysing PC3 Cell-Derived lEVs Metabolites: Four Extraction Methods Compared Using pHILIC and C18

Prior to conducting EV metabolomic analyses, the optimisation of metabolite extraction methodologies is pivotal. Many previous metabolomic studies utilised different metabolite extraction methods for EVs derived from various sources. Clos-Garcia et al. and Zhao et al. employed the methanol–chloroform method, highlighting significant metabolites in PCa and PCa-associated fibroblasts (CAFs), respectively [8,17]. Altadill et al. used the same method for endometrial cancer (EC) patients and PANC1 cell lines [14], while Vallabhaneni et al. used methanol-only extraction for mesenchymal stem cell (MSC)-derived EVs [9]. In contrast, Luo et al. employed a tailored method with 50% methanol and freeze–thaw cycles for pancreatic cancer serum EVs [10]. These variations underscore the need for methodological standardisation in metabolomic investigations. In this study, to evaluate the efficacy of metabolite extraction process from lEVs, four distinct approaches (M-UF, MB-UF, M-F, and MB-F) were tested using two liquid chromatography columns (pHILIC and C18). A Venn diagram was employed to illustrate comparisons, highlighting the varying quantities of extracted metabolites between each method. Additionally, a separate statistical analysis (unpaired *t*-test) was performed to assess the significance of each compared set.

**Figure 2:**
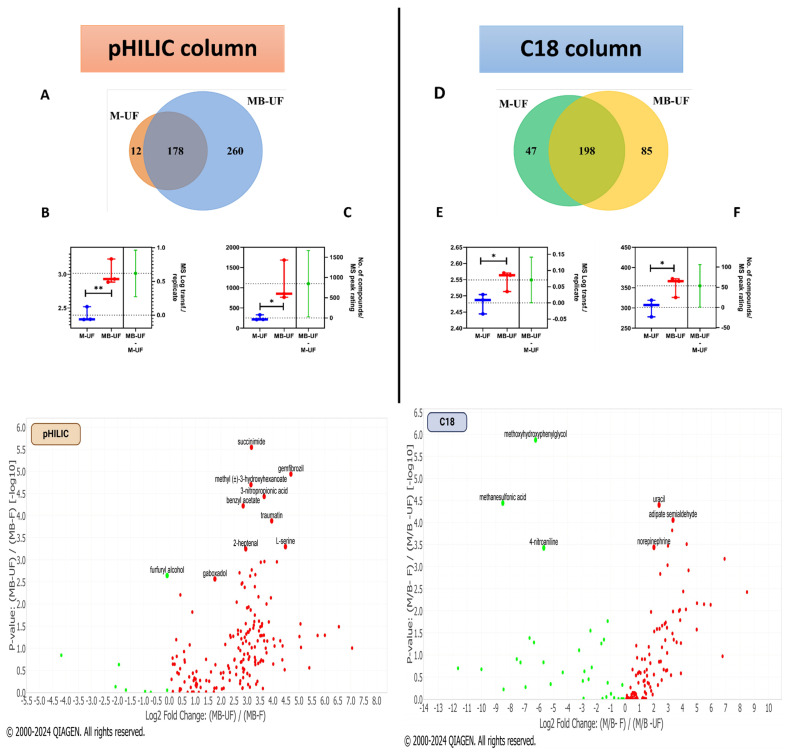
Comparative analysis of metabolite extraction methods using pHILIC and C18. (**A**,**D**) Venn diagrams showing the number of metabolites extracted by each approach using the pHILIC and C18 columns, respectively. (**B**,**E**) MS peak rating LOG transformation per each replicate showing the robustness of each metabolite extraction method employed using the pHILIC and C18 columns, respectively. (**C**,**F**) Number of compounds per each MS peak rating detected after each metabolite extraction approach using the pHILIC and C18 columns, respectively. Using the pHILIC column, most of the metabolites are significantly upregulated, such as gemfibrozil, 3-nitropropionic acid, L-serine, and succinimide, exhibiting fold changes of 4.73, 4.69, 4.48, and 3.25, respectively. Utilising C18, norepinephrine exhibited a positive fold change (2.01), indicating upregulation, while heptanoic acid, mevalonic acid, and methoxyhydroxyphenylglycol showed downregulation, displaying negative fold changes of −8.46, −7.28, and −6.23, respectively. Red dots represent upregulated compounds, whereas green dots indicate downregulated compounds. Error bars denoted ± standard error of the mean (SEM), with significance levels marked as * *p* < 0.05, ** *p* < 0.01. Abbreviations: M-UF, methanol-unfiltered; MB-F, methanol and beads-filtered; MB-UF, methanol and beads-unfiltered; MS, mass spectrometry.

A comparative analysis between the M-UF and MB-UF methods was conducted using three replicates of lEVs to assess the impact of incorporating microbeads in metabolite extraction. For the pHILIC column, the MB-UF method yielded 438 metabolites, surpassing M-UF’s 190, with 178 overlapping. Statistically significant differences (*p* < 0.05) were observed in mass spectrometry (MS) peak rating for total metabolites and compound MS peak rating values (*p* < 0.0076) between the two methods (Figure 2A–C). Similarly, the C18 column showed MB-UF (283 metabolites) outperforming M-UF (245 metabolites), with 198 overlapping, and significant differences in MS peak ratings (*p* < 0.05) (Figure 2D–F). These results suggest the efficacy of microbead utilisation with methanol in extracting a higher quantity of metabolites, underscoring its robustness in identifying compounds aligned with the ChemSpider cloud database.

An additional comparison introduced a filtration step (F) following two metabolite extraction methods (M and MB) to evaluate its significance. Statistical analysis showed no significant differences in MS peak ratings for total metabolites or MS peak values, suggesting that the filtration step may not be critical in the extraction process (Supplementary Materials, Figure S2). Further comparisons (M-UF vs. M-F and MB-UF vs. MB-F) using both columns also revealed no significant differences in MS peak ratings (Supplementary Materials, Figure S3). Consistent data across different separation columns reinforced method reliability. In a final comparison, the pHILIC and C18 columns showed relatively similar total metabolite numbers (476 vs. 462), with 102 overlapping, but no significant differences in MS peak ratings were observed between columns, emphasising methodological consistency (Supplementary Materials, Figure S4). Additionally, the robustness of four tested metabolite extraction approaches across two separation chromatography columns was assessed based on the percentage of identifications for metabolites with a coefficient of variation (CV) of ≤20%. These findings indicate that the highest robustness was achieved by the MB-UF approaches utilising the pHILIC column, with 59.06% of identifications meeting the ≤20% CV criterion. Consistently, MB-UF also showed the highest robustness on the C18 column, with 26.19% of identifications (Table S3). Collectively, the integration of microbeads with methanol demonstrated a notable efficacy in the extraction of metabolites from lEVs. However, the initial findings suggest that the filtration step for the extracted samples does not hold substantial significance. Both the pHILIC and C18 columns displayed effectiveness in metabolite separation. Remarkably, the MB-UF approach showed a unique ability to yield a more extensive array of metabolites; thereby, this would enhance the efficiency of the biomarker discovery process in forthcoming studies.

### 3.3. Identifying Key Metabolites: Comparison of pHILIC and C18 Chromatography

To visualise and interpret differential expression patterns between different experimental conditions, volcano plots (v-plots) were employed to represent the statistical significance identified in metabolite profile–relevant changes with each metabolite extraction approach utilised in this study. In the context of pHILIC column analysis, v-plots revealed a notable increase in the quantity of upregulated metabolites when utilising MB-UF in comparison to MB-F (Figure 2). It is noteworthy that most of the metabolites were significantly upregulated, such as gemfibrozil, 3-nitropropionic acid, L-serine, and succinimide. Consistent with the observed trend, gemfibrozil and succinimide exhibited upregulation when employing M-F in contrast to MB-F (Supplementary Materials, Figure S5). In the case of the C18 column, when comparing the MB-UF to the M-UF extraction method, norepinephrine exhibited downregulation, while metabolites showing upregulation included heptanoic acid, mevalonic acid, and 2,4-dihydroxy-1,4-benzoxazinone (Supplementary Materials, Figure S6). Contrary to this finding, when comparing the MB-F and MB-UF extraction methods, norepinephrine exhibited upregulation, while heptanoic acid and mevalonic acid were identified as downregulated compounds (Figure 2).

In summary, while similarities existed in the metabolic profiling of upregulated and downregulated compounds across different metabolite extraction methodologies, notable differences were observed. The MB-UF approach, especially when employing pHILIC as a separation column, revealed a higher abundance of upregulated compounds compared to other methods, highlighting its potential for reproducibility and efficacy in biomarker discovery in EV metabolomic research. Conversely, using C18 column chromatography with various extraction methods did not show a clear trend in the abundance of upregulated compounds, indicating the absence of a singular superior approach. It is noteworthy that, despite the simplicity and productivity of the MB-UF method, an ingrained limitation remains. The MB-UF method may exhibit selectivity for certain metabolite sets, potentially introducing biases. Despite this, the method provides a comprehensive “snapshot” of metabolites and warrants broader application to better define the pathophysiology of EV marker-related PCa diagnosis over time. Notably, our optimised method, utilising methanol with cell-shearing microbeads, is less destructive and preserves both metabolites and proteins. This allows for the simultaneous exploration of both analytes within the same patient sample, thus making good use of valuable clinical samples.

Prioritising biomarkers with elevated measurements is essential for improving diagnostic accuracy and clinical decision-making. Specifically, selecting approaches that yield a greater abundance of upregulated compounds is paramount, as this enhances the efficacy of biomarker discovery endeavours in subsequent projects. Therefore, it is imperative to align the selection of the metabolite extraction method and separation column with this principle. Bodaghi et al. emphasize the importance of biomarkers showing sensitivity and specificity for disease detection [18], particularly with elevated levels enhancing diagnostic capabilities. Biomarker distinctiveness hinges on the magnitude of alterations, with elevated measurements often indicating disease presence or progression. Amplifying biomarker signals above baseline levels improves detection, especially in low-concentration samples, providing valuable prognostic information. The Food and Drug Administration (FDA) guidelines highlight biomarkers’ utility throughout the disease process, from progression to treatment response evaluation. Moreover, according to Waddell et al., elevated biomarker measurements enable early disease detection, emphasizing their critical role in timely diagnosis and intervention [19]. This underscores the preference for the MB-UF metabolite extraction method and pHILIC separation column in future biomarker discovery endeavours, supported by their practical advantages, including simplicity, feasibility, and cost-effectiveness in EV metabolomic research.

### 3.4. Finding Metabolic Pathways in PCa: Comparison of pHILIC and C18 Chromatography

**Figure 3:**
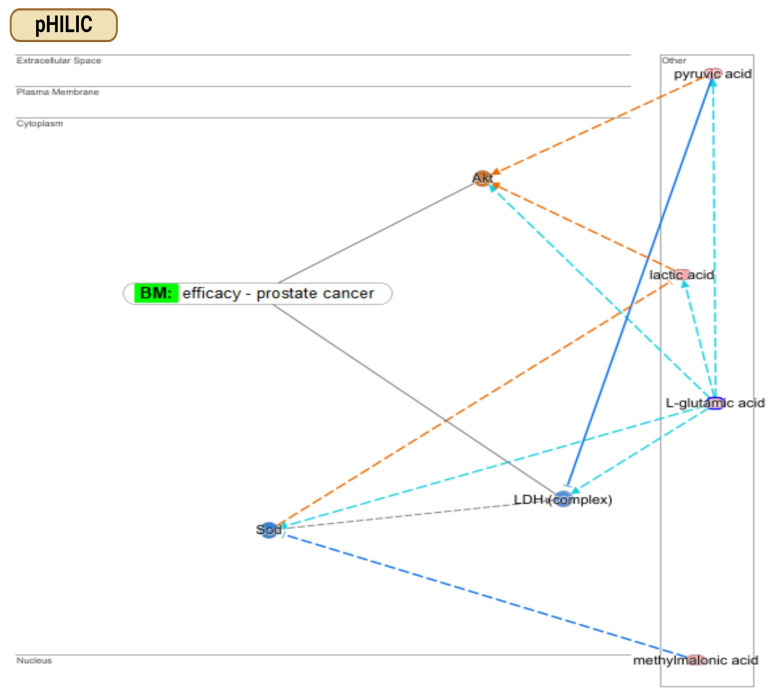

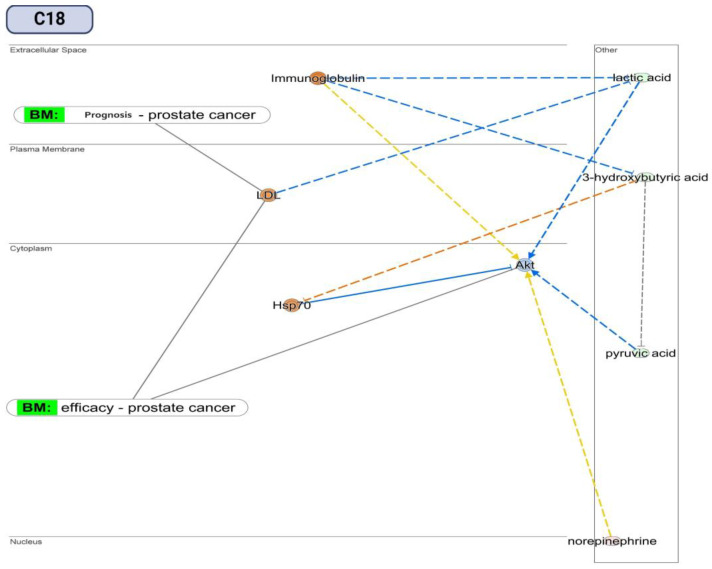
Uncovering metabolite connections within PC3-derived lEVs and their implications in PCa management. In the case of pHILIC, pyruvic acid, lactic acid, L-glutamic acid, and methylmalonic acid emerge as key metabolites, demonstrating both direct and indirect regulatory interactions with cytoplasmic proteins such as Akt and LDH complex. L-glutamic acid accelerates the rate of oxidation of pyruvic acid, consequently activating Akt while inhibiting LDH. Similarly, lactic acid indirectly activates Akt, a process facilitated by L-glutamic acid. Additionally, methylmalonic acid indirectly contributes to the activation of LDH complex through the inhibition of Sod. In the case of C18, lactic acid is depicted as an activator of the Akt protein. The immunoglobulin complex is implicated directly or indirectly in Akt activation, with immunoglobulin promoting the phosphorylation of both lactic acid and 3-hydroxybutyric acid. While 3-hydroxybutyric acid induces pyruvic acid activation, it also triggers HSP 70 inhibition, consequently leading to Akt protein inhibition. These identified metabolites exert both direct and indirect regulatory effects on the Akt protein in the cytoplasm; however, the data suggest a significant trend towards inhibition rather than activation. This finding significantly influences subsequent biomarker discovery processes. Note: Predictions of activation are illustrated by orange arrows, predictions of inhibition by blue arrows, and controversial findings are represented by yellow arrows. Compounds with elevated measurements are represented by pink circles, while green circles represent diminished levels.

To reinforce the findings delineated in the preceding sections, we employed the Ingenuity Pathway Analysis (IPA) QIAGEN software to delineate enriched metabolic pathways exhibiting substantial overlap with metabolites identified individually by the pHILIC and C18 columns. These data are visualised through bar charts and compounds–networks figures generated via the IPA platform (Figure 3). Using the pHILIC column, we identified key metabolic pathways across four metabolite extraction methods (Supplementary Materials, Figure S7 and Table S1). Among the top 10 pathways, metabolites like L-glutamic acid, pyruvic acid, lactic acid, and methylmalonic acid emerged as key regulators in PCa management. Specifically, L-glutamic acid was involved in pathways such as G alpha (q) Signalling Events and Glutamate Dependent Acid Resistance, impacting Akt protein phosphorylation [20]. The Akt protein serves as a biomarker in assessing treatment efficacy, as seen in clinical studies on sirolimus [21] and ridaforolimus [22] in PCa treatment. Additionally, L-glutamic acid influences LDH complex activation [23], linked to PCa prognosis [24] and treatment effectiveness of sipuleucel-T [25]. Significantly, all these metabolites exhibited upregulation upon utilising the MB-UF approach for metabolite extraction, as highlighted (Supplementary Materials, Table S1). Our findings align with a previous EV metabolomic study that reported the upregulation of L-glutamic acid (glutamate) and L-glutamine (glutamine) in a PC3 PCa cell line compared to a PNT2 normal prostate epithelial cell line [26]. According to the KEGG pathways, these metabolites are involved in Arginine Biosynthesis and Alanine, Aspartate, and Glutamate Metabolism. Our results suggest that the metabolites identified from PC3 lEVs mirror the metabolic changes in original PCa cells, supporting their use in cancer biomarker discovery. All in all, an optimal biomarker should yield augmented measurements rather than diminished ones, thereby mitigating potential confusion arising from a low detection limit. This fact reinforces the reliability of the MB-UF approach for subsequent biomarker discovery analyses.

In the context of the C18 column, numerous identified metabolites, including lactic acid, pyruvic acid, and norepinephrine, play roles in activating the human Akt protein [27,28,29] (Figure 3). As previously noted, the Akt protein has been a focal point in numerous clinical trials for monitoring PCa drug efficacy [21,22]. Additionally, the data suggest a potential activation of low-density lipoprotein (LDL), a lipoprotein known to downregulate lactic acid expression [30]. Notably, LDL serves as a reliable biological marker for PCa prognosis and recurrence [31], and it has also been utilised in clinical trials to monitor the effectiveness of “sunitinib” in PCa treatment [32]. In terms of metabolic pathways identified utilising the C18 column, pyruvic acid is implicated in pathways such as Alanine Metabolism, Alanine Degradation III, and Alanine Biogenesis II. Meanwhile, both lactic acid and pyruvic acid participate in the HIF-1α Signalling and White Adipose Tissue Browning pathways (Supplementary Materials, Figure S8 and Table S2).

In conclusion, this study highlights the potential of coupling pHILIC separation with the MB-UF approach to reveal metabolomic signatures relevant to PCa pathogenesis and treatment. The use of pHILIC columns and MB-UF emerges as a promising strategy for biomarker discovery in future metabolomic endeavours, shedding light on disease mechanisms and therapeutic options. Addressing a gap in research, our study comprehensively evaluates diverse metabolite extraction and chromatography separation methods from EVs, offering optimised protocols and aiming to standardise methodologies for consistency across studies. The efficacy of our optimised protocol for biomarker discovery is validated, positioning it for future EV metabolomic investigations involving PCa cell lines and clinical samples.

As for future directions and applications, a large-scale biomarker discovery utilising the optimised protocol would promise novel metabolomic signatures for early diagnosis and prognosis of PCa. The potential of this methodology for clinical translation could facilitate non-invasive diagnostic tests and monitoring tools, enhancing patient management and treatment outcomes. Standardising metabolite extraction and chromatography separation methods will ensure reproducibility and reliability across studies; potentially, the application of this approach could yield mechanistic insights into PCa pathogenesis and therapeutic responses. Additionally, this protocol is also suitable for other cancers, fostering interdisciplinary research through integration with other omics platforms, and supporting longitudinal clinical trials to monitor metabolic changes in PCa patients over time. This study thus paves the way for significant advancements in EV metabolomics and PCa research.

## Data Availability

All data are available on request.

## Supplementary Materials

The following supporting information can be downloaded at: https://www.mdpi.com/article/10.3390/metabo14070367/s1, Figure S1: Characterisation of isolated EVs subpopulation released by PC3 cell line; Figure S2: Comparison between M-F and MB-F approaches regarding the number of metabolites extracted by each method using two columns (pHILIC and C18); Figure S3: Two comparisons including (M-UF versus M-F) and (MB-UF versus MB-F) for the number of metabolites extracted by each method using two columns (pHILIC and C18); Figure S4: Comparison between pHILIC and C18 columns regarding the number of metabolites extracted by four distinct extraction approaches (as whole); Figure S5: Differential expression analysis of compounds using different metabolite extraction methods: a comparison of M-F and MB-F utilising pHILIC chromatography; Figure S6: Comparative volcano plot analysis of compound differential expression with MB-UF versus M-UF extraction methods using C18 chromatography column; Figure S7: Bar chart analysis of enriched canonical pathways associated with metabolites identified via pHILIC separation column; Figure S8: Exploration of enriched canonical pathways linked to identified metabolites using C18 separation column; Table S1: Top enriched metabolic pathways linked to identified metabolites in datasets via pHILIC column; Table S2: Identification of dominant metabolic pathways associated with detected metabolites in datasets through C18 column analysis; Table S3: Percentage of identification for metabolites ≤20% coefficient of variation (CV) for four distinct metabolite extraction approaches across two chromatography columns.
