# Supplementary material for: Optimising Extracellular Vesicle Metabolomic Methodology for Prostate Cancer Biomarker Discovery
Source: Metabolites. 2024 Jun 28;14(7):367. doi: 10.3390/metabo14070367 (PMC11279087; doi:10.3390/metabo14070367)
Supplement: Supplementary file 1 [file metabolites-14-00367-s001.zip › metabolites-3065439-supplementary.pdf]

Supplementary Data

Optimising Extracellular Vesicle Metabolomics Methodology for Prostate Cancer Biomarker Discovery

Supplementary methods

Protein extraction and quantification

EV samples were subjected to lysis utilising a lysis buffer supplemented with a protease inhibitor cocktail at a ratio of 100:1 (Sigma-Aldrich Pty Ltd, NSW, Australia). Subsequently, the EV lysates were subjected to oscillation for a duration of 30 min at a temperature of 4 °C, followed by a 15-second sonication treatment performed on ice. The lysates were then subjected to centrifugation at 14,000 × g for a duration of 15 min at 4 °C. The resulting supernatants were carefully collected and subsequently stored at a temperature of -80 °C for preservation. A BCA protein assay kit (Thermo Scientific) was harnessed to ascertain the protein content within the isolated EV samples, following strict adherence to the instructed manufacturer's guidelines. In the endeavour to establish a comprehensive standard curve encompassing a spectrum of 0-200 µg/mL, a series of seven serial dilutions were accurately prepared employing bovine serum albumin (BSA). A 1:10 dilution of the sample was accomplished by utilising distilled water, yielding a final volume of 150 µL. This diluted sample was subsequently combined with an equivalent volume (150 µL) of the BCA working solution. Both the samples and the standard dilution serial were diligently replicated (three replicates), followed by an incubation period of 2 hours (h) at a temperature of 37°C. For quantitative assessment, the absorbance of each sample was measured employing a microplate reader, specifically the Synergy HT system (Bio-Tek), operating at a wavelength of 562 nm. The recorded absorbance values were subsequently converted to µg/mL units via reference to the established standard curve. The ultimate protein concentration was then adjusted by multiplication with the respective dilution factor (1:10), yielding the precise protein content present within the EV isolates.

Western blotting (WB)

In the context of WB analysis, a uniform quantity of protein (ranging from 10 to 20 µg) was diligently segregated from analogous samples. These protein aliquots were subjected to separation on a 4-12%

Bis-Tris protein gel (Bio-Rad, USA), after which they were carefully transferred onto polyvinylidene fluoride (PVDF) membranes with a pore size of 0.22 µm (Millipore/Merck). Following the transfer, the PVDF membrane underwent blocking with a 5% BSA solution in TBST buffer over a duration of 1 h at ambient RT. Sequentially, the membrane was exposed to primary antibodies overnight (o/n) at a temperature of 4 °C. The concentrations of the primary antibodies were diluted as per the recommendations provided by the respective manufacturers. Following three consecutive 5-minute washes with TBST, the membrane was subsequently subjected to a 1-h incubation at room temperature (RT) with a secondary antibody conjugated with horseradish peroxidase (HRP) from Thermo Fisher Scientific. This was succeeded by another set of three 5-minute washes utilising TBST. The visualisation of immunoblot bands was achieved through the utilisation of enhanced chemiluminescence (ECL) western blotting substrate (SuperSignal West Dura, Thermo Fisher Scientific). The imaging of the immunoblot bands was facilitated using the ChemiDoc Imaging System (Bio-Rad).

Antibodies for WB

Antibody	Company
**Goat anti-human ApoB Mab, FITC**	Abcam, Cat. No. ab27637
**Goat IgG FITC**	Abcam, Cat. No. ab37374
**Mouse IgG1 APC**	Thermo Scientific, Cat. No. MA518093
**Mouse anti-human CD63 MAb, APC**	Thermo Scientific, Cat. No. A15712
**Mouse anti-human CD81 MAb, APC**	Thermo Scientific, Cat. No. 349510
**Rabbit anti-human Calnexin MAb**	Abcam, Cat. No. ab133615
**Rabbit anti-human Flotillin 1**	Abcam, Cat. No. ab133497
**Rabbit anti-human HSP70 MAb**	Abcam, Cat. No. ab181606
**Rabbit anti human Syntenin Mab**	Abcam, Cat. No. ab133267

Global metabolomics using ZIC-pHILIC and C18 separation columns.

On the experiment day, dry metabolite-containing samples of different metabolite-extraction approaches were resuspended in 50 µL of CH3CN:H2O (9:1) (v/v) (PH 5.5). The samples were injected into liquid chromatography (LC) using a ZIC-pHILIC column (150 × 64.6 mm, 5 mm) (Sigma, Millibrore) for separation. LC-MS analysis was conducted using a UHPLC system with a Q-Exactive HF mass spectrometer, employing both positive and negative ion modes. MS/MS data was acquired using DDA mode, and various parameters such as spray voltages, gas settings, and temperatures were applied. A separation technique was employed utilising mobile phases as defined in Table 2.1 at a flow rate of 300 µL/min. The gradient started at 20% B and was held for 2 min, increased to 40% B over 19 min, then went to 100% B for 1 min, and returned to 0% B for 9 min for re-equilibration. The mass spectrometer, with a heated electrospray ionization probe, operated in data-dependent mode, scanning from 75 to 1000 mass range at 60K resolution. The top 20 ions were subjected to MS/MS at 15K resolution with positive and negative spray voltages. An instrument calibration preceded data acquisition for optimal analysis. In case of C18 column (150 × 64.6 mm, 5 mm) (Sigma, Millibrore), similar instrument adjustment was applied, whereas dry metabolite-containing samples of different metabolite-extraction approaches were resuspended in 50 µL of 0.1 formic acid (PH 3).

Summary of mobile phases employed by different columns for metabolite separation.

Column	Buffers	Mode tested	Separation
**C18**	CH3CN/H2O in 0.1% formic acid	+ /-	Elution by hydrophobicity
**ZIC-pHILIC**	CH3CN:C3H8O (1:9 v/v), 20 mM NH4CO2	+/-	Elution by pH and polarity and ion exchange
**ZIC-pHILIC plus sample pre-filtered**	CH3CN/H2O 10 mM NH4CO2	-	As above with less ion interaction

**ZIC-pHILIC no pre-filtering**	CH3CN/H2O 10 mM NH4CO2	-	As above with less ion interaction
**ZIC-pHILIC plus sample pre-filtered**	CH3CN/H2O 10 mM $C_2H_7NO_2$ (ammonium acetate)	-	As above with different ion interaction
**ZIC-pHILIC no pre-filtering**	CH3CN/H2O 10 mM $C_2H_7NO_2$ (ammonium acetate)	-	As above with different ion interaction

Supplementary (S) figures

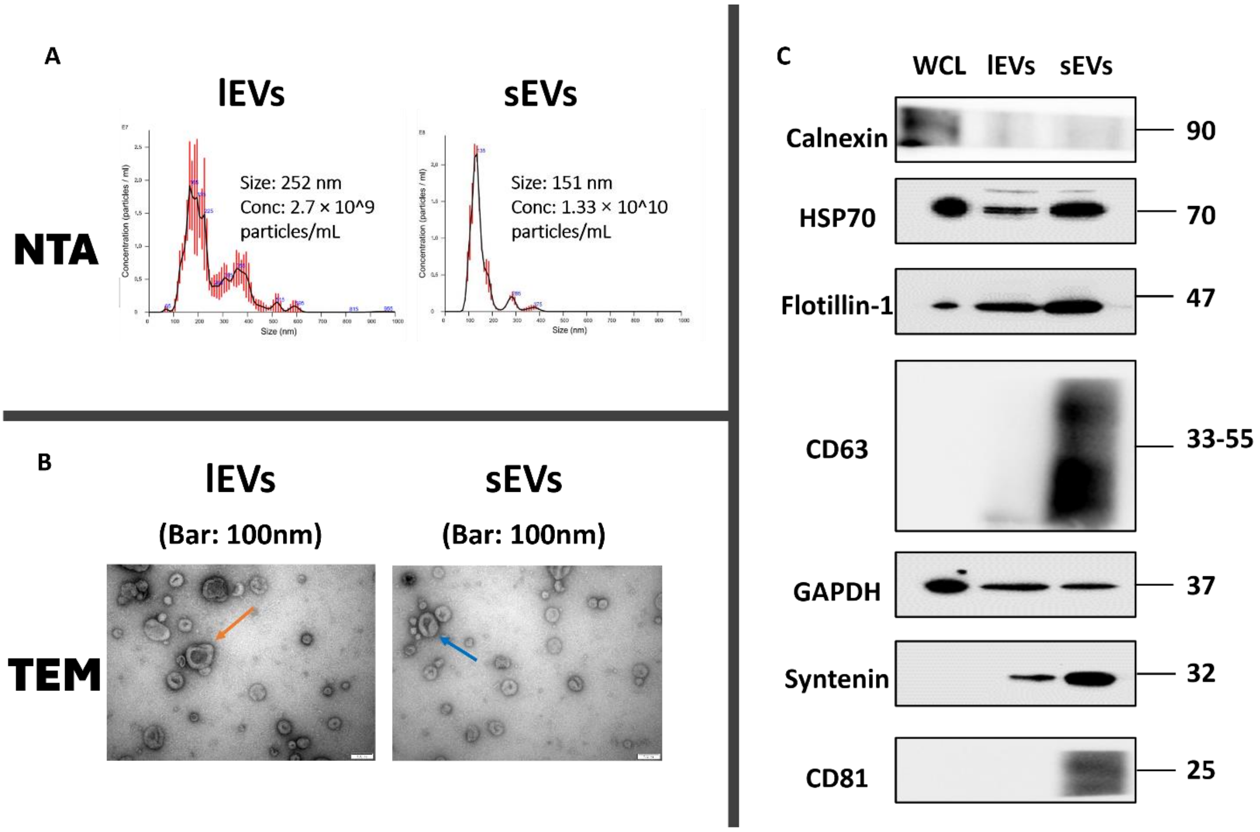

Figure S1: Characterisation of isolated EVs subpopulation released by PC3 cell line. (A) NTA analysis shows the average mean size of EVs. **(B)** TEM examination shows the typical cup-shaped morphology of isolated EVs. **(C)** WB analysis represents EVs positive biomarker, such as CD81, CD63, syntenin, flotillin-1 and HSP70, whereas calnexin used as a negative control. **Abbreviations:** NTA: nanoparticle tracking analysis; lEVs: large extracellular vesicles; sEVs: small extracellular vesicles; TEM; transmission electron microscopy; WCL: whole cell lysate.

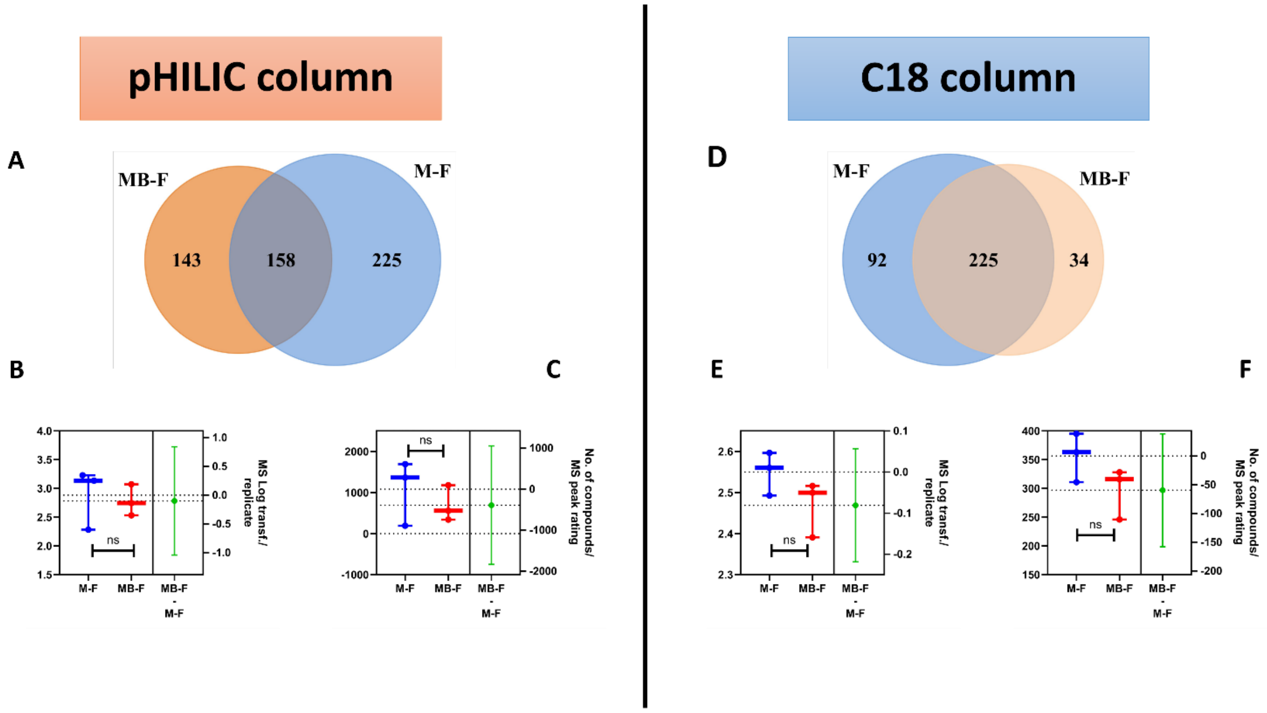

Figure S2: Comparison between M-F and MB-F approaches regarding the number of metabolites extracted by each method using two columns (pHILIC and C18). (A, D) Venn diagrams showing the number of metabolites extracted by each approach using pHILIC and C18 columns, respectively. **(B, E)** MS peak rating LOG transformation per each replicate showing the robustness of each metabolite extraction method employed using pHILIC and C18 columns, respectively. **(C, F)** Number of compounds per each MS peak rating detected after each metabolite extraction approach using pHILIC and C18 columns, respectively. Three replicates (n=3) of PC3-derived lEVs were extracted by each metabolite extraction method separately. Each dot in **B**, **C**, **E** and **F** represents one replicate. The data is presented as mean ± standard error of the mean (SEM), and statistical analysis was performed using (unpaired) two tailed t-test. The significance level was set at (*P<0.05, **P<0.01, ***P<0.001, ****P<0.0001) to indicate the level of statistical significance. **Abbreviations:** M-F methanol-filtered; MB-F, methanol and beads-filtered.

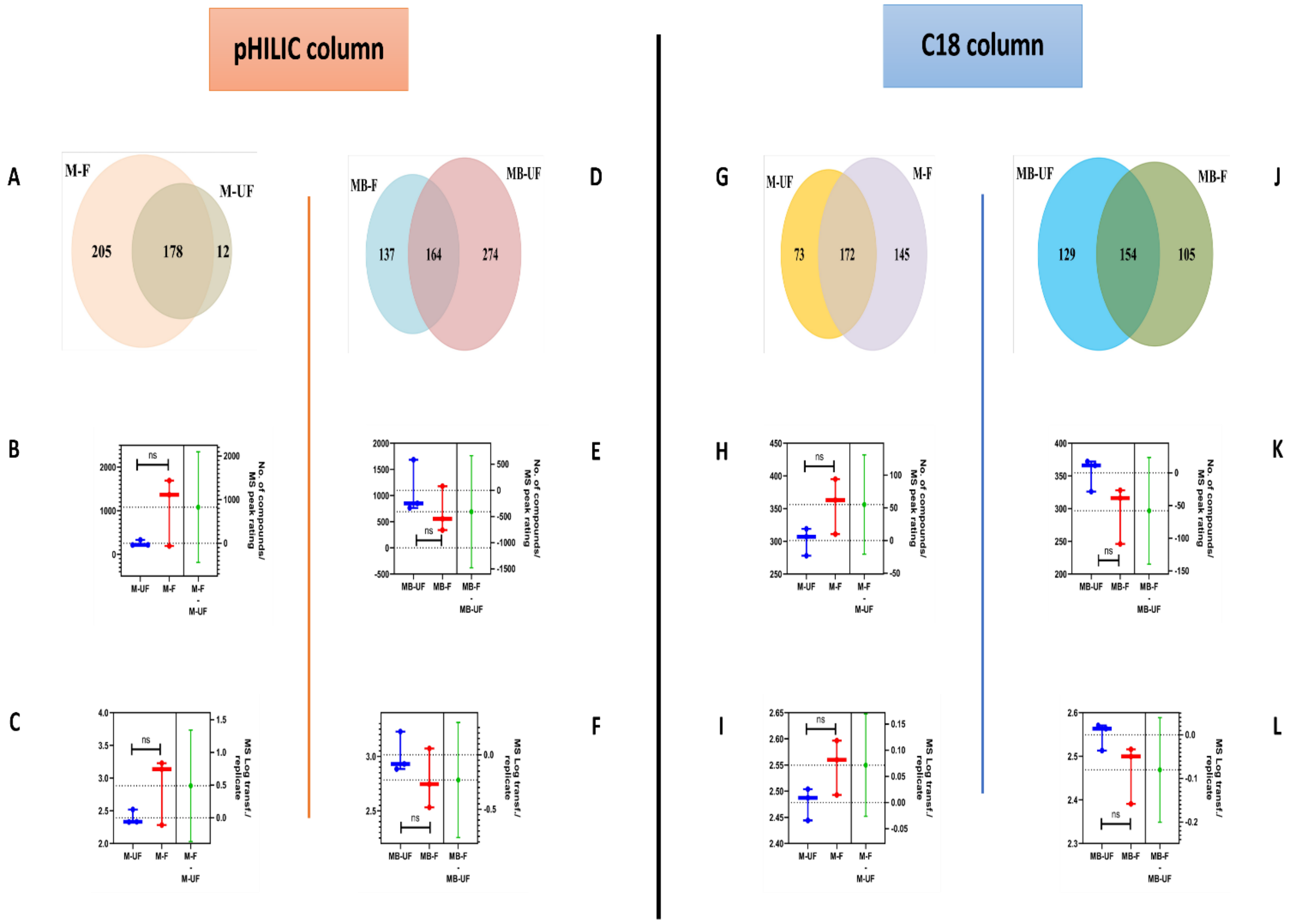

Figure S3: Two comparisons including (M-UF versus M-F) and (MB-UF versus MB-F) for the number of metabolites extracted by each method using two columns (pHILIC and C18). (A, D) and (G, J) Venn diagrams showing the number of metabolites extracted by each approach using pHILIC and C18 columns, respectively. **(B, E) and (H, K)** Number of compounds per each MS peak rating detected after each metabolite extraction approach using pHILIC and C18 columns, respectively. **(C, F) and (I, L)** MS peak rating LOG transformation per each replicate showing the robustness of each metabolite extraction method employed using pHILIC and C18 columns, respectively. Three replicates (n=3) of PC3-derived lEVs were extracted by each metabolite extraction method separately. Each dot in **B, C, E, F, H, I, K and L** represents one replicate. The data is presented as mean± standard error of the mean (SEM), and statistical analysis was performed using (unpaired) two tailed t-test. The significance level was set at (**P*<0.05, ***P*<0.01, ****P*<0.001, *****P*<0.0001) to indicate the level of statistical significance. **Abbreviations:** M-F, methanol-filtered; MB-F, methanol and beads-filtered; M-UF, methanol-unfiltered; MB-UF, methanol and beads-unfiltered.

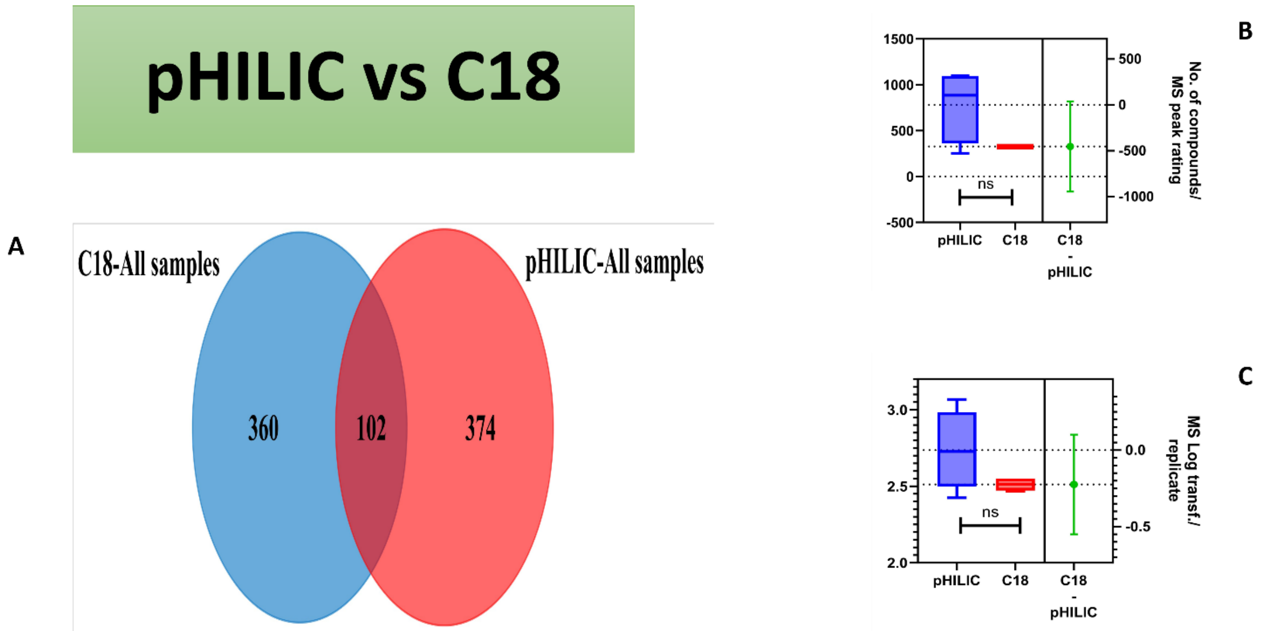

Figure S4: Comparison between pHILIC and C18 columns regarding the number of metabolites extracted by four distinct extraction approaches (as whole). (A) Venn diagram showing the number of metabolites extracted by all metabolite extraction methods using pHILIC and C18 columns. **(B)** Average number of compounds per each MS peak rating detected utilising four different metabolite extraction approaches using pHILIC and C18 columns. **(C)** MS peak rating LOG transformation for all replicates throughout four different extraction methods showing the robustness of each column regarding separation ability. The data is presented as mean± standard error of the mean (SEM), and statistical analysis was performed using (unpaired) two tailed t-test. The significance level was set at (**P*<0.05, ***P*<0.01, ****P*<0.001, *****P*< 0.0001) to indicate the level of statistical significance.

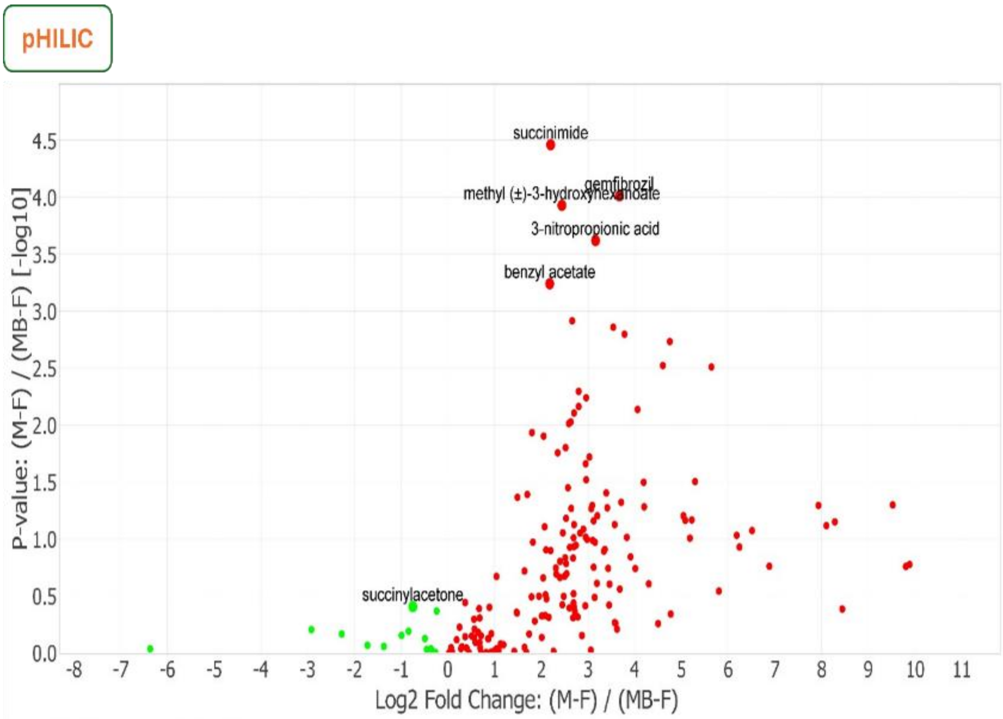

Figure S5: Differential expression analysis of compounds using different metabolite extraction methods: a comparison of M-F and MB-F utilising pHILIC chromatography. Succinimide, gemfibrozil and (±)-3-hydroxynonanoic acid are reported among significantly upregulated metabolites, sharing a similar trend presented by MB-UF approach (mentioned previously). Each point/arrow represents an individual compound; red colour represents upregulated compounds, whereas green colour indicates downregulated compounds. **Abbreviations:** M-F, methanol- filtered; MB-F, methanol and beads-filtered.

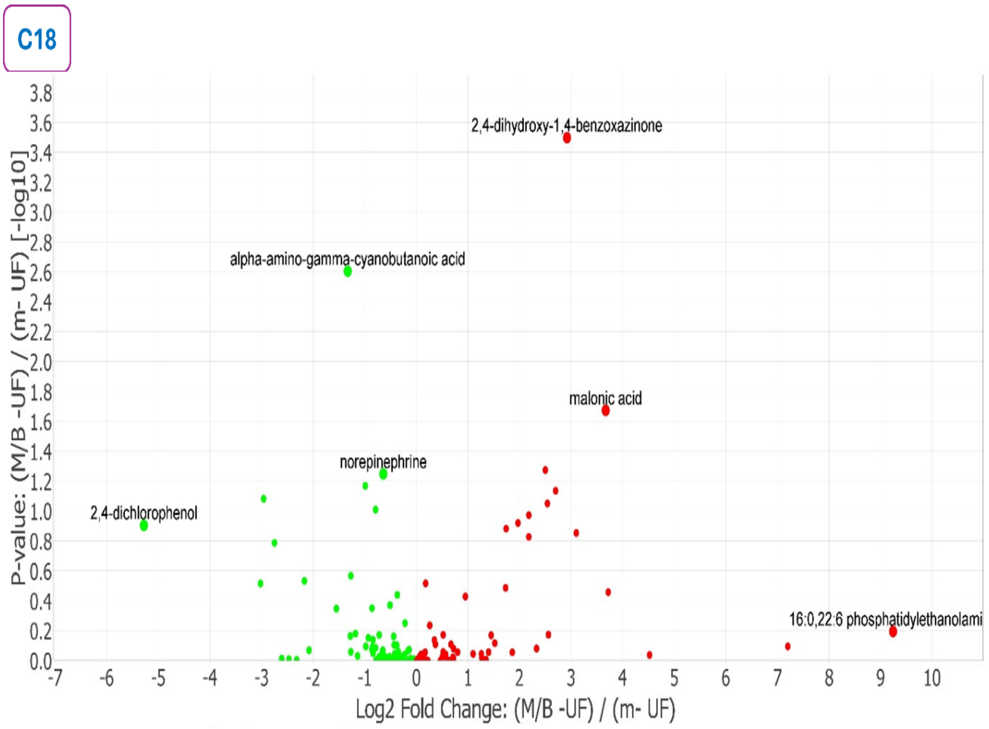

Figure S6: Comparative volcano plot analysis of compound differential expression with MB-UF versus M-UF extraction methods using C18 chromatography column. Utilising the MB-UF extraction method, norepinephrine exhibited downregulation, while malonic acid demonstrated upregulation. Each point/arrow represents an individual compound. Red colour represents upregulated compounds, whereas downregulated compounds are represented by green colour. Thresholds for significance levels were set at fold change (Expression Log ratio ±2) and P<0.05 (-log10 p-value=1.301). **Note:** compounds highlighted by bold font match similar compound IDs in the four cloud databases (HMDB, CAS, KEGG and ChemSpider) employed in this analysis to compare against, while compounds in normal font matches IDs recorded in at least one of these databases. **Abbreviations:** M-UF, methanol-unfiltered; MB-UF, methanol and beads-unfiltered.

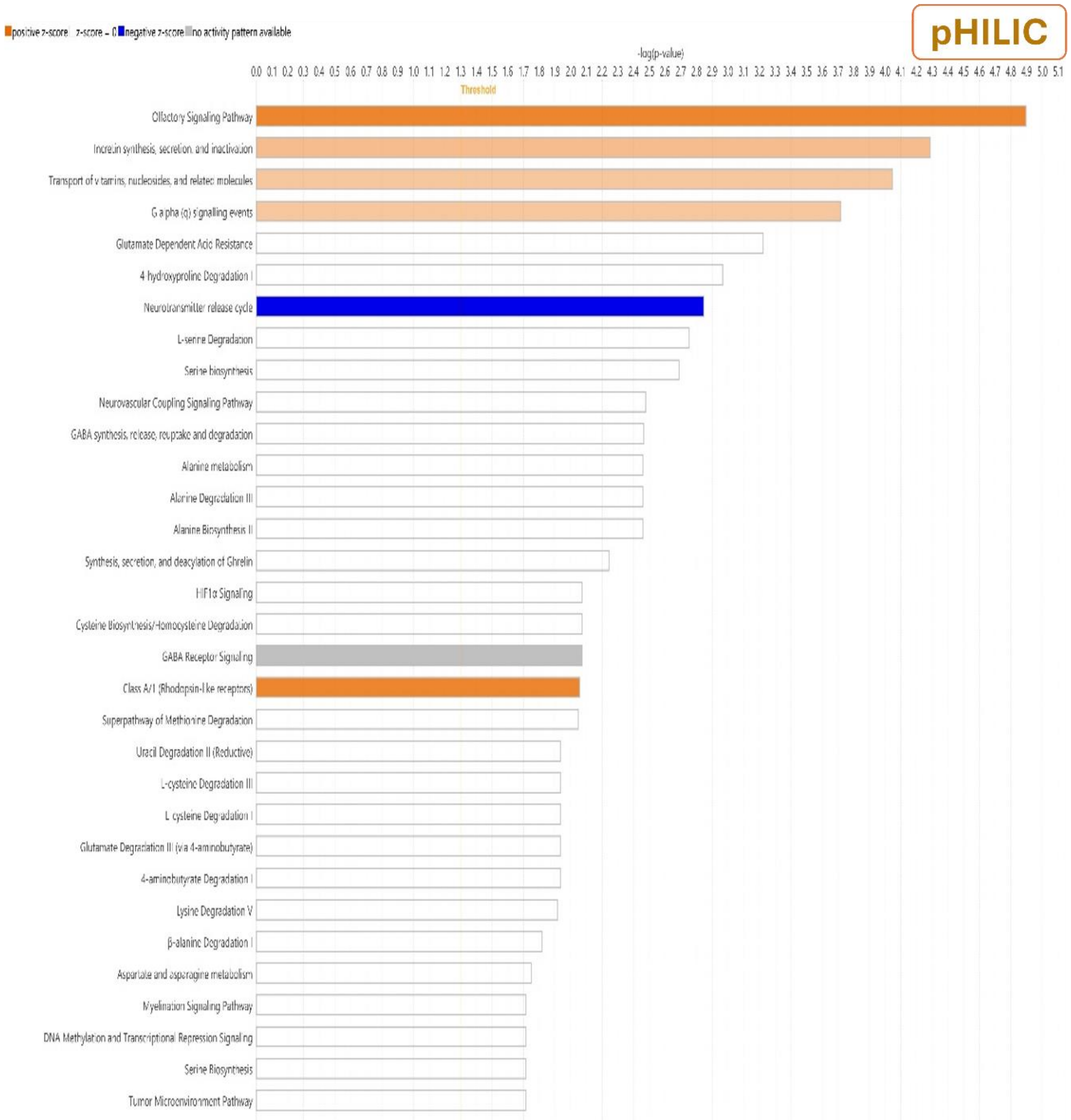

Figure S7: Bar chart analysis of enriched canonical pathways associated with metabolites identified via pHILIC separation column. The pathways are arranged based on their level of significance, with the tallest bars indicating a higher degree of overlap between these pathways and the identified metabolites in the datasets. Significance was determined with a threshold set at a $P<0.05$ (-log10 p-value=1.301). Z-scores were utilised to quantify the predictive capacity for pathway activation/inhibition, with colour intensity reflecting the level of prediction. Positive z-scores depicted by orange bars indicate the prediction of pathway activation, while negative z-scores represented by blue bars signify the prediction of inhibition. A z-score of zero illustrated by white bars denotes equilibrium

between activation and inhibition predictions, while pathways lacking a predefined activity pattern are depicted by grey bars. Metabolites identified through pHILIC column separation demonstrated compelling evidence of activation (upregulation) within the most significantly enriched pathways (top four orange bars), underscoring the potential of pHILIC column in EV metabolomic research for biomarker discovery.

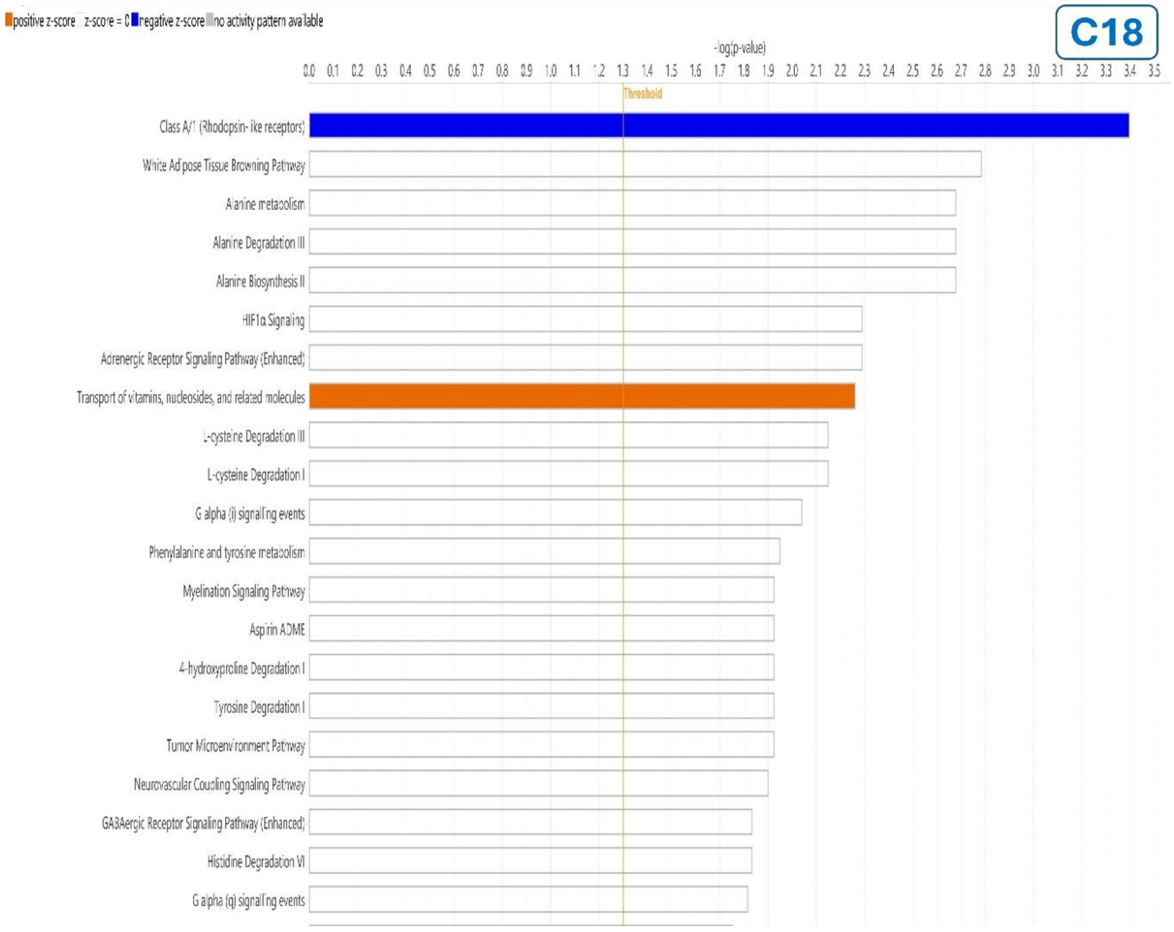

Figure S8: Exploration of enriched canonical pathways linked to identified metabolites using C18 separation column. The height of the bars in the pathways diagram corresponds to the degree of overlap between the pathways and the identified metabolites, with taller bars indicating a higher level of significance. Significance was determined using a threshold of *P*<0.05 (-log10 p-value=1.301). The predictive capacity for pathway activation/inhibition was quantified using z-scores, with colour intensity indicating the level of prediction.

Positive z-scores (orange bars) suggest pathway activation, while negative z-scores (blue bars) indicate pathway inhibition. A z-score of zero (white bars) represents equilibrium between activation and inhibition predictions, while pathways lacking a defined activity pattern are depicted by grey bars. Metabolites identified through C18 column are likely implicated in metabolic pathways exhibiting either zero or negative z-scores, indicating no strong evidence of activation.

Supplementary (S) tables

Table S1: Top enriched metabolic pathways linked to identified metabolites in datasets via pHILIC column.

Metabolic pathways/Metabolites - pHILIC		M-UF vs MB-UF	M-F vs MB-F	M-UF vs M-F	MB-UF vs MB-F
**Olfactory signalling Pathway**	guaiacol	↓	↑	↓	↑
	heptanoic acid	↑	↑	↓	↑
	hexanoic acid	↑	↑	↑	↑
	isovaleric acid	↑	↑	↑	↑
	lauric acid	↓	↑	↑	↑
	octanoic acid	↓	↑	↑	↑
**Incretin synthesis, secretion and activation**	lauric acid	↓	↑	↑	↑
	myristic acid	↓	↑	↑	↑
	palmitic acid	↑	↑	↓	↑
	pentadecanoic acid	↑	↑	↑	↑
	stearic acid	↑	↑	↓	↑
**Transport of vitamins and nucleotides**	cytosine	↑	↑	↑	↑
	decanoic acid	↓	↑	↓	↑
	lauric acid	↓	↑	↑	↑
	myristic acid	↓	↑	↑	↑
	palmitic acid	↑	↑	↓	↑
	stearic acid	↑	↑	↓	↑
	thymine	↓	↑	↑	↑

	uracil	↑	↑	↑	↑
**G alpha (q) signalling events**	hexanoic acid	↑	↑	↑	↑
	L-glutamic acid	↑	↑	↓	↑
	lauric acid	↓	↑	↑	↑
	myristic acid	↓	↑	↑	↑
	palmitic acid	↑	↑	↓	↑
	pentadecanoic acid	↑	↑	↑	↑
	stearic acid	↑	↑	↓	↑
**Glutamate dependant acid resistance**	GABA	↑	↑	↑	↑
	L-glutamic acid	↑	↑	↓	↑
**4-hydroxyproline degradation I**	L-erythro-4-hydroxyglutamate	↓	↑	↓	↑
	L-glutamic acid	↑	↑	↓	↑
	pyruvic acid	↓	↑	↓	↑
**Neurotransmitter release cycle**	L-glutamic acid	↑	↑	↓	↑
	lauric acid	↓	↑	↑	↑
	myristic acid	↓	↑	↑	↑
	palmitic acid	↑	↑	↓	↑
	stearic acid	↑	↑	↓	↑
**L-serine degradation**	L-serine	↓	↑	↑	↑
	pyruvic acid	↓	↑	↓	↑
**Serine biogenesis**	L-glutamic acid	↑	↑	↓	↑
	L-serine	↓	↑	↑	↑
	pyruvic acid	↓	↑	↓	↑
**Neurovascular coupling signalling pathway**	GABA	↑	↑	↑	↑
	L-glutamic acid	↑	↑	↓	↑
	lactic acid	↑	↑	↑	↑
	pyruvic acid	↓	↑	↓	↑

Notes: red arrows represent upregulation, while downregulation is represented by green arrows.
Abbreviations: F, filtered; M, methanol; MB, methanol and beads; UF, unfiltered.

Table S2: Identification of dominant metabolic pathways associated with detected metabolites in datasets through C18 column analysis.

Metabolic pathways/Metabolites - C18		MB-UF vs M-UF	MB-F vs M-F	M-F vs M-UF	MB-F vs MB-UF
**Class A/1 (Rhodopsin-like receptors)**	2-oxoglutaric acid	↓	↓	↑	↓
	3-hydroxybutyric acid	↑	↑	↓	↑
	3-hydroxyoctanoic acid	↓	↓	↑	↑
	norepinephrine	↓	↑	↑	↑
	oleic acid	↓	↑	↑	↑
	pentanoic acid	↓	↓	↑	↑
	stearic acid	↑	↓	↑	↓
**White adipose tissue browning pathway**	lactic acid	↓	↑	↓	↑
	norepinephrine	↓	↑	↑	↑
	pyruvic acid	↓	↑	↓	↑
**Alanine metabolism**	2-oxoglutaric acid	↓	↓	↑	↓
	pyruvic acid	↓	↑	↑	↑
**Alanine degradation III**	2-oxoglutaric acid	↓	↓	↑	↓
	pyruvic acid	↓	↑	↓	↑
**Alanine biogenesis II**	2-oxoglutaric acid	↓	↓	↑	↓
	pyruvic acid	↓	↑	↓	↑
**HIF 1 α signalling**	lactic acid	↓	↑	↓	↑
	pyruvic acid	↓	↑	↓	↑
**Adrenergic receptor signalling pathway**	dopa	↓	↓	↑	↑
	methoxyhydroxyphenylglycol	↓	↓	↓	↓
	norepinephrine	↓	↑	↑	↑
**Transport of vitamins and nucleosides**	cytosine	↓	↑	↑	↑
	norepinephrine	↓	↑	↑	↑
	oleic acid	↓	↑	↑	↑
	stearic acid	↑	↓	↑	↓
	uracil	↑	↑	↑	↑
**L-cysteine degradation III**	2-oxoglutaric acid	↓	↓	↑	↓
	pyruvic acid	↓	↑	↓	↑
**L-cysteine degradation I**	2-oxoglutaric acid	↓	↓	↑	↓
	pyruvic acid	↓	↑	↓	↑

**G alpha (i) signalling events**	2-oxoglutaric acid	⬇	⬇	⬆	⬇
	3-hydroxybutyric acid	⬆	⬆	⬇	⬆
	3-hydroxyoctanoic acid	⬇	⬇	⬆	⬆
	norepinephrine	⬇	⬆	⬆	⬆

Notes: red arrows represent upregulation, while downregulation is represented by green arrows.
Abbreviations: F, filtered; M, methanol; MB, methanol and beads; UF, unfiltered.

Table S3: Percentage of identification for metabolites ≤20% coefficient of variation (CV) for four distinct metabolite extraction approaches across two chromatography columns.

	M-F	M-UF	MB-F	**MB-UF**
**C18**	16.93	24.83	25.96	26.19
**pHILIC**	21.17	5.20	5.65	59.06

Abbreviations: F, filtered; M, methanol; MB, methanol and beads; UF, unfiltered.
